# Fatal Herniotomy Due to Abnormal Obturator Arteries

**Published:** 1880-09

**Authors:** 


FATAL HERNIOTOMY DUE TO ABNORMAL OBTURATOR ARTERIES.

The following cases are reported as occurring in the Sussex County Hospital:

Case I was that of a woman aged 62, who had a swelling in the left groin for four months. When first seen she was in a collapsed condition, with dry, brown tongue, and frequent stercoraceous vomiting; pulse 100; feeble. Her expression was anxious; she complained of pain at the umbilicus; there was a small hard swelling at the left femoral ring, without impulse on coughing. A few hours after admission, herniotomy was performed. A small knuckle of intestine was found very tightly constricted at Gimbernat's ligament; the ligament was incised, but, before the bowel could be reduced, a second stricture, formed by the sac itself, had to be divided. When Gimbernat's ligament was divided, free bleeding occurred, but this was apparently controlled without difficulty. The day after the operation, some hemorrhage was detected from the wound, and as it could not be controlled, the wound was again opened and the bleeding point searched for, but without success. The patient gradually sank, and died on the third day after the operation. At the necropsy the neighborhood of the wound of incision was found infiltrated with blood, which extended beneath the peritoneum as high as the umbilicus. The hemorrhage was found to proceed from the complete division of the obturator artery, which arose from the external iliac in common with the epigastric, and crossed over the femoral sheath and along the inner margin of the crural ring.

Case II was that of a woman aged 64, who had had a rupture five or six years, but had always been able to return it herself. She had never worn a truss. On admission, four days after her inability to return it, there was a hard swelling, about the size of a walnut, in the position of the femoral ring, without impulse, and irreducible. Soon after admission, she vomited a material, exactly resembling thick liquid fæces in appearance and smell. Herniotomy was performed under anæsthetic mixture. A small knuckle of bowel and a piece of omentum, which were found in the sac in a somewhat congested condition, were returned without difficulty, only a very slight nick in Gimbernat's ligament being needed to relieve the stricture. No hemorrhage occurred at the time of operation. She was apparently much relieved by the operation, and passed a good night, but the following evening she began to complain of a frequent desire to micturate, and of some tenderness of the abdomen, and on the day but one after the operation she showed signs of severe collapse, and vomiting recommenced. The collapse increased, and she died early the following morning. There was no hemorrhage from the wound after the operation. At the necropsy, eight hours after death, the wound was found healthy in appearance externally, without any ecchymosis of the surrounding skin, but on opening it out some clotted blood appeared, and on tracing this back, a large mass of clot was found in the subperitoneal tissues surrounding the wound, and in the neighboring parts of the pelvis. It was found that the obturator artery, which arose from the external iliac in common with the epigastric and passed over the crural ring on its course to the pelvis, had been partially divided, probably at the time when Gimbernat's ligament was nicked. The obturator artery on the opposite side did not arise from the external iliac, and was probably normal in origin. The reporter added that the interest of the two cases centered, of course, in the abnormal distribution of the obturator artery, in each case the cause of death being its accidental division, which was in the one case partial, in the other complete.—*Medical Record.*

